# Antioxidant Nanomaterial Based on Core–Shell Silica Nanospheres with Surface-Bound Caffeic Acid: A Promising Vehicle for Oxidation-Sensitive Drugs

**DOI:** 10.3390/nano9020214

**Published:** 2019-02-06

**Authors:** Francisco Arriagada, Germán Günther, Jaume Nos, Santi Nonell, Claudio Olea-Azar, Javier Morales

**Affiliations:** 1 Departamento de Ciencias y Tecnología Farmacéuticas, Facultad de Ciencias Químicas y Farmacéuticas, Universidad de Chile, Santiago 8380494, Chile; farriagadajs@ug.uchile.cl; 2 Departamento de Química Orgánica y Fisicoquímica, Facultad de Ciencias Químicas y Farmacéuticas, Universidad de Chile, Santiago 8380494, Chile; ggunther@ciq.uchile.cl; 3 Institut Químic de Sarrià (IQS), University Ramon Llull, Via Augusta 390, 08017 Barcelona, Spain; jaimenosa@iqs.url.edu (J.N.); santi.nonell@iqs.url.edu (S.N.); 4 Departamento de Química Inorgánica y Analítica, Facultad de Ciencias Químicas y Farmacéuticas, Universidad de Chile, Santiago 8380494, Chile; caolea@ciq.uchile.cl

**Keywords:** silica nanoparticles, core–shell, singlet oxygen, caffeic acid, polyphenols, nanocarrier, antioxidant

## Abstract

The design of efficient, biocompatible, and easily prepared vehicles for drug delivery is a subject of great interest for medicine and pharmaceutical sciences. To achieve the above goals, surface functionalization is critical. Here, we report a hybrid nanocarrier consisting of core–shell silica nanospheres and the antioxidant caffeic acid linked to the surface, to evaluate their in vitro antioxidant capacity, their capability to protect oxidation-sensitive compounds incorporated in nanoparticles, and to study the interaction with bovine serum albumin protein. The results show that the radical-scavenging activity of immobilized caffeic acid is attenuated in the silica nanospheres; however, other antioxidant properties such as Fe^2+^-chelating activity and singlet oxygen quenching are enhanced. In addition, caffeic acid is protected from binding to proteins by the nanoparticle, suggesting that this nanosystem is more likely to maintain the antioxidant activity of caffeic acid in biological media. Finally, the natural antioxidant barrier on the nanocarrier is able to delay the degradation of a compound incorporated into this nanovehicle. Considering all findings, this work proposes a suitable tool for pharmaceutical and cosmetic industries as an antioxidant nanocarrier for oxidation-sensitive drugs.

## 1. Introduction

The design of efficient, biocompatible, and easily prepared vehicles for drug delivery is a subject of great interest for medicine and the pharmaceutical sciences [1]. Since Vallet-Regí et al. [2] reported the use of mesoporous silica nanoparticles (SNPs) as drug delivery systems, porous silica-based materials were extensively studied [3,4,5,6], because they are inert, versatile, and mechanically stable materials. The advance in these materials offers different types, shapes, and sizes depending on the desired purpose. In particular, core–shell materials have attractive properties such as higher density or mechanical durability [7,8], and they also serve as a template for the potential selective removal of the dense core in order to obtain a nanocapsule system. 

Additionally, their surface can be easily functionalized in order to meet the needs of a controlled-release agent, to enhance the stability of the nanoparticle, to make them more biocompatible, and to improve their targeting capacity, among others [9,10,11,12,13]. To achieve the above goals, many molecules [14,15,16,17], including natural antioxidant compounds [18,19], were used as surface modifiers.

Natural antioxidants, such as polyphenols, present in several plants and their products [20] attracted great attention since they were related with many health benefits in the treatment of various pathologies [21]. They were also used to improve the cosmetic properties of several formulations [22,23] and delay the deterioration of products in the food industry [24]. Despite their attractive properties and the wide scope of their potential applications, the use of natural antioxidants is often limited, due to their high sensitivity to environmental factors such as light, oxygen, pH, and others [25,26,27,28]. In addition, it was reported that polyphenols interact with some proteins, reducing their antioxidant activity [29]. To alleviate these problems, some authors used them co-condensed on silica particles [30] or simply adsorbed [31]. However, the interactions between the antioxidant molecules themselves and between the antioxidants and the silica surface can reduce their antioxidant activity, depending on the moiety or molecular scaffold involved [30,31,32,33,34]. 

An alternative approach is the covalent grafting of antioxidants on the surface of silica materials. Reports of such antioxidant materials indicate that the presence of antioxidants on the silica surface reduces its toxicity [35] and improves the stability of the antioxidant, as well the hybrid material, which can be reused after washing without affecting its antiradical properties [36]. These approaches propose a promising biocompatible carrier; however, further evaluation of its antioxidant properties is necessary. To our knowledge, no reports exist on (1) the ability of silica-bound antioxidants to quench singlet oxygen, a non-radical reactive oxygen species [37], and (2) the ability of antioxidant nanomaterials to protect incorporated drugs.

We set out to study whether immobilization of polyphenols onto silica nanoparticles could form a barrier capable of protecting an incorporated drug from oxidative environments.

Therefore, the goals of this work were (1) to develop and characterize an antioxidant nanomaterial based on the caffeic acid covalently linked onto a core–shell silica nanosphere, (2) to evaluate its in vitro antioxidant capacity as an antiradical (2,2-diphenyl-1-picryl-hydrazyl (DPPH^●^)), metal-chelating (Fe^2+^) agent, and a singlet oxygen quencher, and (3) as a proof of concept, to evaluate its ability to protect an oxidation-sensitive molecule incorporated into the nanoparticles.

## 2. Materials and Methods 

### 2.1. Materials

Caffeic acid (CA, ≥98%), *N*-hydroxysuccinimide (NHS, 98%), *N*-(3-dimethylaminopropyl)-*N*′-ethylcarbodiimide hydrochloride (EDC, ≥98%), tetraethyl orthosilicate (TEOS, 98%), (3-aminopropyl)triethoxysilane (APTES, ≥98%), hexadecyltrimethylammonium chloride solution (CTAC, 25 wt.%), 3-Å molecular sieves, anhydrous dichloromethane (DCM, ≥99,8%), 5,6-diphenyl-3-(2-pyridyl)-1,2,4-triazine-4′,4′′-disulfonic acid sodium salt (ferrozine, ≥97%), iron(II) chloride tetrahydrate (FeCl_2_·4H_2_O, 98%), 2,2-diphenyl-1-picryl-hydrazyl (DPPH^●^), 3-(triethoxysilil)propyl isocyanate (95%), 4,7,10-trioxa-1,13-tridecanediamine (97%), and rose bengal (95%) were purchased from Sigma-Aldrich. Bovine serum albumin (BSA fraction V, ≥98%), ethanol (HPLC grade), fuming hydrochloric acid (HCl, ACS reagent 37%), acetonitrile (HPLC grade), methanol (HPLC grade), and triethylamine (TEA, ≥99%) were obtained from Merck. Ammonium hydroxide (NH_4_OH, ACS reagent 28–30%) and tetrahydrofuran (THF, ≥99.8%) were purchased from J.T. Baker, while 9,10-anthracenedipropionic acid disodium salt (ADPA) was purchased from AdipoGen Chemodex (Buckingham, United Kingdom). Deionized water (Milli-Q, 18.2 MΩ·cm) was used in all experiments in this study. All materials were used as received.

### 2.2. Preparation of Mesoporous Core–Shell Silica Nanospheres (CSSNs)

CSSNs were obtained in two steps according to previously reported procedures with slight modifications [38,39,40]. Firstly, in order to synthesize a solid core, ethanol (44 mL), deionized water (3 mL), and ammonium hydroxide (1.3 mL) were added to a round-bottomed flask and stirred for 10 min. Later, TEOS (1.73 mL) was added to the mixture and allowed to react for 3 h under stirring (850 rpm) at room temperature. The nanoparticles formed were collected by centrifugation (15,000× *g*), washed with water and ethanol several times, and finally dried and stored until further use. 

In the second step, 540 μL of 25 wt.% CTAC solution was dissolved in a mixture of 30 mL of water, 30 mL of ethanol, and 550 μL of NH_4_OH; the mixture was then stirred for 5 min, and mixed with 100 mg of solid-core nanoparticles suspended in 20 mL of water. Finally, 250 μL of TEOS was added and left to react for 4 h at room temperature. The product was then washed with water and ethanol, and treated with 60 mL of an acid ethanolic solution (1.2 M HCl final concentration) for 18 h at 80 °C to remove the CTAC. This procedure was carried out twice to ensure complete removal of CTAC. Finally, the product was washed with water and ethanol, and is herein called core–shell silica nanospheres (CSSNs).

### 2.3. Preparation of Amino-Functionalized Core–Shell Silica Nanospheres (ACSSNs)

Firstly, 100 mg of the above-prepared CSSNs was suspended in 20 mL of ethanol at 40 °C, and 500 μL of APTES was added. The mixture was stirred for 12 h at 40 °C, and the modified nanoparticles were then centrifuged and washed with ethanol. The resulting nanoparticles (ACSSNs) were dried and stored until further use.

### 2.4. Conjugation of Caffeic Acid to Amino-Functionalized Core–Shell Silica Nanospheres (ACSSNs-CA)

Grafting of caffeic acid (CA) to ACSSNs was achieved by coupling its –COOH group to the –NH_2_ groups of ACSSNs using the EDC/NHS coupling agents. Briefly, 20 mg of CA, 42.6 mg of EDC, and 51.2 mg of NHS were dissolved in water, and the mixture was sonicated for 15 min. Then, the mixture was added dropwise to a suspension of 100 mg of ACSSNs, and the reaction was allowed to proceed under stirring for 6 h at room temperature. The resulting product was collected by centrifugation and washed three times with ethanol to remove by-products and unreacted reagents. The resulting nanoparticles (ACSSNs-CA) were dried and stored until further use.

### 2.5. Characterization

The hydrodynamic diameter of the nanoparticles suspended in ethanol was measured with dynamic light scattering (DLS) at 25 °C, using a Malvern Zetasizer Nano ZS90 (Malvern, UK) with a detection angle of 173° and an equilibration time of 120 s. Each measurement was performed three times. The zeta potentials were obtained using the same equipment at 25 °C with the nanoparticles suspended in 1 mM NaCl aqueous solution. For size analysis using scanning transmission electron microscopy (STEM), the samples were dispersed in ethanol, before a 10-μL aliquot was deposited onto a silicon grid, dried for 24 h, and then coated with gold. TEM images were taken on an FEI^TM^ inspect F50 model microscope, with an accelerating voltage of 10.00 kV. Fourier-transform infrared (FT-IR) spectra were obtained using an Interspec 200-X FT-IR spectrometer with 4-cm^−1^ resolution, between in 4000 and 400 cm^−1^, and the final spectrum corresponds to an average of 16 scans. The thermogravimetric analysis (TGA) was performed using a Netzsch TG 209 F1, under air flow of 20 mL·min^−1^ with a heating rate of 10 °C·min^−1^ in the range of 20–800 °C.

Determination of CA grafting onto nanoparticles was also quantified using a previously validated HPLC indirect method. Chromatographic analysis was performed on an Agilent series 1100 (Agilent Technologies, Baden-Württemberg, Germany) using a Hypersil ODS C18 (4.6 × 250 mm, 5-µm particle size) column from Agilent. Gradient elution was performed using a mobile phase consisting of a mixture of phase A (0.01% orthophosphoric acid solution, pH 4.2) and phase B (acetonitrile). Firstly, the starting mobile phase was 90% A and 10% B for 7 min; then, from 8 to 14 min, an isocratic elution mixture of 70% A and 30% B was used, pumped at a flow rate of 1.0 mL/min. The sample injection volume was 10 µL, the run time was 14 min, and the retention time was 12.3 min. A PDA detector was set at 325 nm. The HPLC standard curve (*y* = (5.749 × 10^6^)*x* + 22.28) was obtained by plotting the area values vs. caffeic acid concentration, and was linear (*R^2^* = 0.9994) within the range of 1.25 × 10^−4^ to 2.25 × 10^−3^ M in ethanol. Data were obtained from triplicate analysis. 

### 2.6. Antioxidant Capacity Evaluation

#### 2.6.1. DPPH^●^ Radical Assay

The DPPH^●^ radical assay was performed according to previously reported methods, with slight modifications [41]. Briefly, 1 mL of a DPPH^●^ 50 µM solution freshly prepared in methanol was mixed in a 5-mL volumetric flask with solutions of CA, either free or nanoparticle-bound, reaching a final concentration range of 10–100 µM. The samples were stirred in the dark for 20 min at 25 °C, then centrifuged, and the supernatant was measured spectrophotometrically at 515 nm. A control using CA-free nanoparticles under the same conditions was measured. The radical-scavenging activity (% RSA) was calculated according to Equation (1).

(1) $$\%RSA=\left(\frac{A_{0}-A_{1}}{A_{0}}\right)\;\times \;100$$

where *A*_0_ is the control absorbance and *A*_1_ is the sample absorbance.

#### 2.6.2. Measurements of Chelating Activity

The chelating capacities of the nanomaterials (ACSSNs-CA) and free CA were investigated based on their ability to inhibit the formation of the Fe^2+^–ferrozine complex, expressed as a percentage (%) of Fe^2+^-chelating activity, using the method described by Dinis et al. [42] and G. Berlier et al. [43]. In a 5-mL volumetric flask, different concentrations of nanomaterials or free CA (10−100 μM) were mixed with 100 μL of FeCl_2_ (2 mM) and 200 μL of ferrozine (2 mM) in an ethanol/acetate buffer solution (15/85 *v*/*v*, pH 5.0) and stirred for 10 min, allowing the samples to reach equilibrium. After centrifugation, the absorbance of the supernatant was measured at 562 nm. A control using CA-free nanoparticles under the same conditions was measured. The chelating activity, expressed as a percentage, was calculated according to Equation (2).

(2) $$\%\;Chelating\;activity=\left(\frac{A_{0}-A_{1}}{A_{0}}\right)\;\times \;100$$

where *A*_0_ is the absorbance of the solution containing only FeCl_2_ and ferrozine, and *A*_1_ is the absorption of the sample containing either polyphenol or nanomaterials.

#### 2.6.3. Singlet Oxygen Quenching

Direct detection of ^1^O_2_ near-infrared phosphorescence at 1275 nm was performed using a customized PicoQuant Fluotime 200 lifetime system [44]. A diode-pumped pulsed Nd:YAG laser (CryLas, FTSS355-Q, Berlin Germany) working at a 1-kHz repetition rate at 532 nm was used for excitation. The time-resolved ^1^O_2_ emission data were fitted to Equation (3) [45] using GraphPad Prism 5 software.

(3) $$S\left(t\right)=S_{0}\frac{\tau_{\Delta }}{\tau_{\Delta }-\tau_{T}}\left(e^{-\frac{t}{\tau_{\Delta }}}-e^{-\frac{t}{\tau_{T}}}\right)$$

where *S(t)* is the ^1^O_2_ signal intensity at time *t*, *S_0_* is an empirical parameter proportional to singlet oxygen quantum yield, *τ_Δ_* is the ^1^O_2_ lifetime, and *τ_T_* is the triplet-state lifetime [45]. The total quenching rate constant (*k*_q_) for the deactivation of ^1^O_2_ by ACSSNs-CA or free CA dispersed in ethanol was obtained by measuring the first-order rate of singlet oxygen luminescence decay in the presence and absence of a quencher, and using rose bengal (RB) electrostatically adsorbed onto amino-functionalized silica nanoparticles as a sensitizer when the nanomaterial was evaluated. A control using CA-free nanoparticles under the same conditions was measured. The *k*_q_ values were calculated from the slope of the Stern–Volmer plots, according to Equation (4).

(4) $$\tau^{-1}=\tau_{0}^{-1}+k_{q}\left[Q\right]$$

where *τ^−1^* and *τ_0_^−1^* are the singlet oxygen lifetimes in the presence and absence of a quencher (*Q*), respectively [45].

### 2.7. Reaction of Singlet Oxygen with Anthracene Dipropionic Acid (ADPA)

ADPA was chosen as a target model for the evaluation of the protection conferred by the CA barrier in the nanoparticles. To this end, a short-length silylated ADPA derivative and a long-chain linker (for later attachment of CA) were prepared following the protocols reported by Bresolí-Obach et al. [46]. Both derivatives were then grafted onto CSSNs and, finally, CA was attached to the long-chain linker.

Briefly, 4 mg of ADPA, 14.8 mg of EDC, and 14.6 mg of NHS were dissolved in dry CH_2_Cl_2_ with drops of THF as a co-solvent. The solution was stirred for 60 min in a cold bath and added dropwise to a stirred solution of 4 µL of APTES solution in CH_2_Cl_2_. The reaction was left stirring at room temperature overnight. The mixture was subsequently used without further purification. Similarly, 220 µL of 4,7,10-trioxa-1,13-tridecanediamine and 83 µL of 3-(triethoxysilyl)propyl isocyanate were mixed in 2 mL of ethanol and allowed to react for 24 h at room temperature. The mixture was diluted with 3 mL of ethanol and subsequently used without further purification.

Functionalization of the CSSNs with both silylated derivatives was achieved by adding dropwise a mixture of 100 mg of CSSNs suspended in acetonitrile. The mixture was stirred for 24 h at room temperature and then centrifuged and washed several times. A fraction of the nanoparticles were further functionalized with CA using the same procedure as for ACSSNs-CA. The two series of ADPA-containing nanoparticles were named nano-ADPA and nano-ADPA-CA, respectively.

Ethanolic solutions containing 3 μM ADPA (either as nano-ADPA or as nano-ADPA-CA) and 3 μM rose bengal (RB), the singlet oxygen photosensitizer, were irradiated at 524 nm, and the reaction of ADPA with singlet oxygen was monitored recording the anthracene fluorescence intensity on a Fluoromax-4 spectrofluorometer.

### 2.8. Interaction of the Antioxidant Nanomaterial with Bovine Serum Albumin (BSA)

Measurements were made on a Cary Eclipse fluorescence spectrophotometer (Agilent Technologies, CA, USA) using a rectangular 1.0-cm path-length quartz cuvette. Emission spectra of tryptophan were recorded in the range of 300–500 nm. The excitation wavelength was 280 nm, with both slits fixed at 5 nm. The experiments were carried out following the methodology described by Li et al., with slight modifications [47]. Although CA has a valley at the excitation wavelength (molar absorptivity lower than 5000 M^−1^·cm^−1^), its concentration was nevertheless kept low (below 30 µM) to minimize its absorption. Likewise, the tryptophan emission was monitored at 380 nm, where caffeic acid does not absorb, to avoid inner filter effects.

Different sample aliquots (ACSSNs-CA or free CA) from stock solutions containing 1.34 mM CA were mixed with 2.5 mL of BSA (4 μM) and diluted to 10.0 mL with 1 mM phosphate-buffered saline (pH 7.4). The samples were kept at 25 °C for 15 min and the fluorescence intensity was recorded in three independent experiments.

Fluorescence deactivation was estimated using the Stern–Volmer model, according to Equation (5) [48].

(5) $$\frac{F_{0}}{F}=1+k_{q,BSA}\tau_{0}\left[Q\right]=1+K_{SV}\left[Q\right]$$

where *F*_0_ and *F* are the fluorescence intensities at 380 nm in the absence and presence of the quencher, respectively, *k*_q,BSA_ is the bimolecular deactivation constant, *τ_0_* is the lifetime of the fluorophore in the absence of the quencher, *K*_SV_ is the fluorescence Stern–Volmer constant, which is a measure of the deactivation efficiency, and *[Q]* is the quencher concentration.

The binding constants and the binding sites, assuming a static deactivation mechanism, can be estimated using the double-logarithm model, according to Equation (6).

(6) $$log\frac{F_{0}-F}{F}=logK_{a}+n\;log\left[Q\right]$$

where *K*_a_ is the binding constant and *n* is the number of BSA binding sites.

### 2.9. Statistical Analysis

All results are expressed as means ± standard deviation (SD) of a least three experiments. Significant differences (* *p* < 0.05; ** *p* < 0.001) were analyzed using a Student’s *t*-test (two groups) or ANOVA (three or more groups) using GraphPad Prism software version 6.012 (La Jolla, California, USA).

## 3. Results and Discussion

### 3.1. Core–Shell Silica Nanospheres

**Figure 1:**
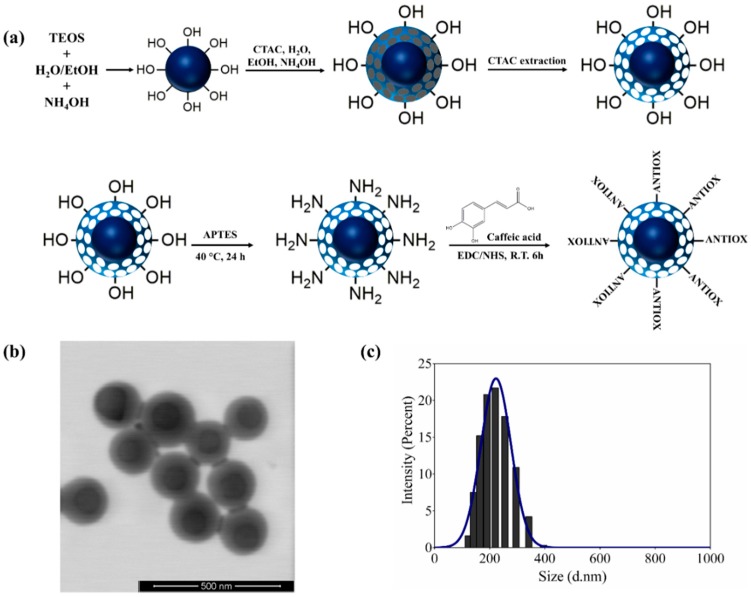
(**a**) Scheme of the process to obtain amino-functionalized core–shell silica nanospheres with immobilized caffeic acid (ACSSNs-CA). (**b**) TEM image of ACSSNs-CA. (**c**) ACSSNs-CA size distribution obtained with dynamic light scattering (DLS) measurements.

Hybrid nanocarriers with antioxidant properties, based on core–shell silica nanospheres and the polyphenol CA, were obtained (Figure 1). A uniform 91-nm dense silica core was synthesized, which was then coated with a mesoporous silica shell using CTAC. This material has the advantage of being a template to potentially remove its core and obtain a nanocapsule with various functions. Subsequently, the surfactant was removed and nanoparticles with a hydrodynamic diameter of 199.1 nm were obtained, which were denominated as core–shell silica nanospheres (CSSNs) (Figure 1a).

The CSSNs were amino-functionalized with APTES (ACSSNs) and, later, the antioxidant caffeic acid was immobilized onto the nanospheres. The coupling agent EDC was used to activate the –COOH group of caffeic acid and to form a further, more stable, intermediate in the presence of NHS that facilitates the reaction with the –NH_2_ group on the amino-functionalized nanoparticle surface, forming an amide bond. This synthesis route has the advantage that the EDC and NHS are easily manipulated, are soluble in aqueous medium and in some organic solvents, and the by-products and excess reagents can be easily removed [49,50]. The nanosystem obtained through the EDC/NHS coupling pathway was designated as ACSSNPs-CA (Figure 1a). 

The results showed an increment in the hydrodynamic diameter of CSSNs after amino-functionalization (ACSSNs), from 199.1 nm to 224.5 nm. The change in the hydrodynamic diameter would suggest the presence of the amino groups on the surface of the CSSNs, but was mainly due to the slight agglomeration of the ACSSNs, as evidenced by the change in the value of the polydispersity index (PdI). Nevertheless, the PdI of all the materials suggests remarkably monodisperse samples.

On the other hand, a minor size change was observed between CSSNs (199.1 nm) and ACSSNs-CA (205.7 nm), which corroborated that the functionalization and subsequent immobilization of caffeic acid did not significantly change the size of the material. The DLS measurements were corroborated by STEM. The ACSSNs-CA TEM image (Figure 1b) shows the spherical geometry of the nanomaterials, where the dense core had an average diameter of 98.6 ± 11.8 nm and the shell had an average thickness of 39.4 ± 6.6 nm. The whole nanosphere had an average diameter of 186.03 ± 14.6 nm. These results are in concordance with the ACSSNs-CA DLS measurements (Figure 1c).

The zeta potential of CSSNs was −32 mV and changed to −3 mV when amino-functionalized, which suggests the successful incorporation of the amino groups (–NH_2_) onto the surface of the nanoparticles and corroborated the slight agglomeration of ACSSNs and the increase in the hydrodynamic diameter. The variation in the zeta potential from −3 mV for ACSSNs to −22.6 mV for ACSSNs-CA suggests a neutralization of the ionizable fraction of the nanoparticles and surface modification due to CA. Table 1 summarizes the hydrodynamic diameter and the zeta potential values for the obtained nanomaterials.

**Figure 2:**
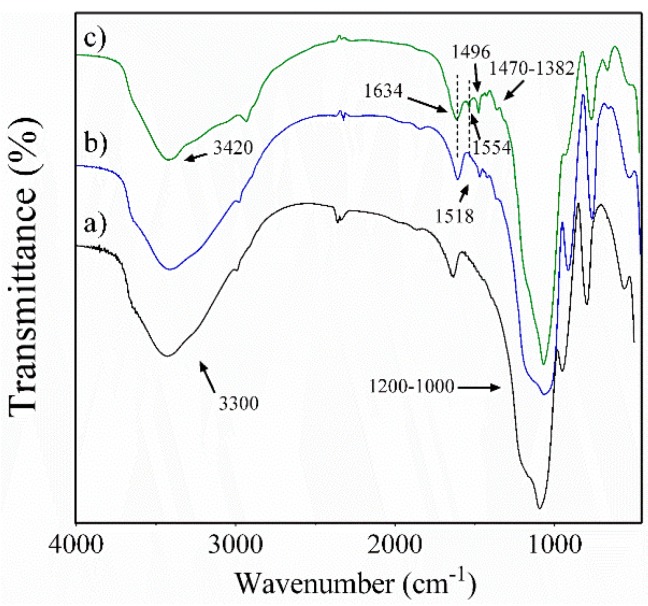
Fourier-transform infrared (FT-IR) curves of (**a**) CSSNs (black line), (**b**) ACSSNs (blue line), and (**c**) ACSSNs-CA (green line).

Moreover, the presence of CA on ACSSNs was confirmed by FT-IR analysis (Figure 2), where typical signals for CSSNs (black line) are 1200−1000 cm^−1^ due to the (≡Si–O–Si≡) groups and 3300 cm^−1^ corresponding to the stretching of the adsorbed water. The ACSSNs spectrum (blue line) showed bands at 2982 cm^−1^ and 2940 cm^−1^, corresponding to (–CH_2_) in the APTES propyl chain, as well as characteristic vibration bands of the free NH_2_ group at 1518 cm^−1^ [31], confirming the successful incorporation of the amino groups onto surface. The ACSSNPs-CA spectrum (green line) showed characteristic bands of amide-bond formation at 1634 cm^−1^ and 1554 cm^−1^ [36]. Furthermore, the spectrum exhibited a characteristic band at 1496 cm^−1^, attributable to aromatic stretching (C=C), and 1448 cm^−1^, 1385 cm^−1^, and 1340 cm^−1^ peaks, corresponding to the OH/CO combination modes and the aliphatic bending (–CH) of CA [43].

Additionally, the quantitation of CA on the surface of the nanospheres was obtained with TGA and confirmed by HPLC analysis (Table 1). 

TGA revealed that, for every 100 mg of nanosphere, 11.77 mg of CA was incorporated. This value was corroborated using an indirect method. After the grafting reaction, the product was washed several times, and the supernatant was filtered and measured using HPLC. The analysis revealed that, for every 100 mg of nanosphere, 12.5 mg of CA was incorporated, and these results are consistent with the TGA values (Table 1). 

The CA-containing nanomaterial obtained via EDC/NHS coupling reactions showed a large amount of CA per gram of product (694 µmol/g). In addition, its handling was simple and was carried out in a few steps, which facilitated its optimization.

### 3.2. Antioxidant Capacity of Nanosystems

Antiradical capacity was measured using the DPPH^●^ radical assay, which is a simple, fast. and reproducible method used as a screening technique for the ability of compounds to scavenge free radicals and also to compare their effectiveness with other studies [51,52]. The test is based on radical DPPH^●^ reduction to non-radical DPPH-H, resulting in a solution color change from deep purple to yellow. Although different mechanisms proposed by other authors depend on factors such as molecule, solvent, and medium structure, it is generally accepted that the reaction between phenols and DPPH^●^ proceeds through the “sequential proton-loss electron-transfer” mechanism [52].

**Figure 3:**
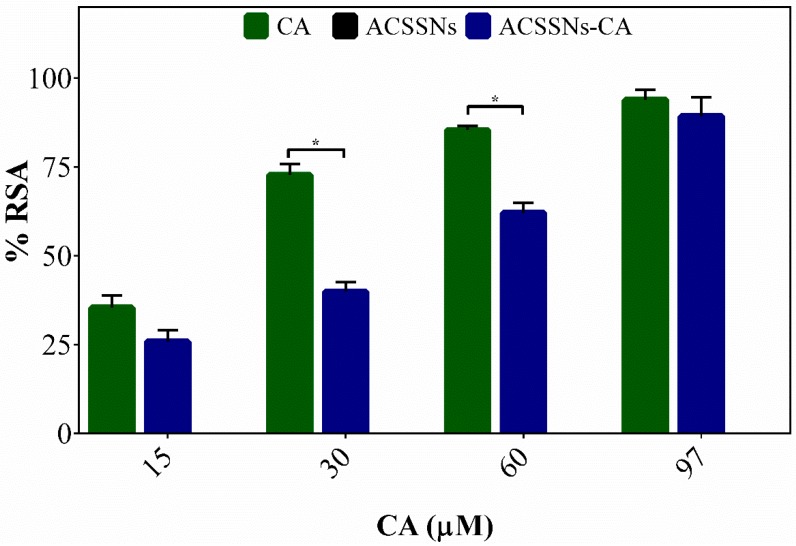
Comparison of percentage radical-scavenging activity (%RSA) between free caffeic acid (CA), caffeic acid grafted onto core–shell silica nanoparticles (ACSSNs-CA), and caffeic-acid-free nanosphere (ACSSNs) with radical 2,2-diphenyl-1-picryl-hydrazyl (DPPH^●^). Results are reported as means ± SD (*n* = 3). * *p* < 0.05.

In the case of CA, the scavenging activity was mainly provided by the catechol group present in the molecule, and the stabilizing effect was provided by the benzene ring, the bridge with a double bond, and the carboxyl group [53,54]. As shown in Figure 3, for ACSSNs-CA, a CA concentration increase in the range of 15–97 μM led to an increase in the radical-scavenging activity, expressed as %RSA. A comparison of the %RSA between CA and ACSSNs-CA showed that the antiradical capacity was directly related to the %CA on the nanoparticle. The results showed an attenuation in the scavenging activity in the nanoparticles when compared to free CA, which was expected due to hindrance caused by the mesoporous nanoparticle surface, since it was involved in the diffusion of the radical species and the interaction between DPPH^●^ and the nanoparticles [36]. As expected, the ACSSNs showed no effect against DPPH^●^. However, the data indicated that CA antioxidant activity was not abolished because it maintained the necessary scaffold to carry out the mechanism; this persistent great antioxidant effect was also reported by other authors [36,43,55]. 

**Figure 4:**
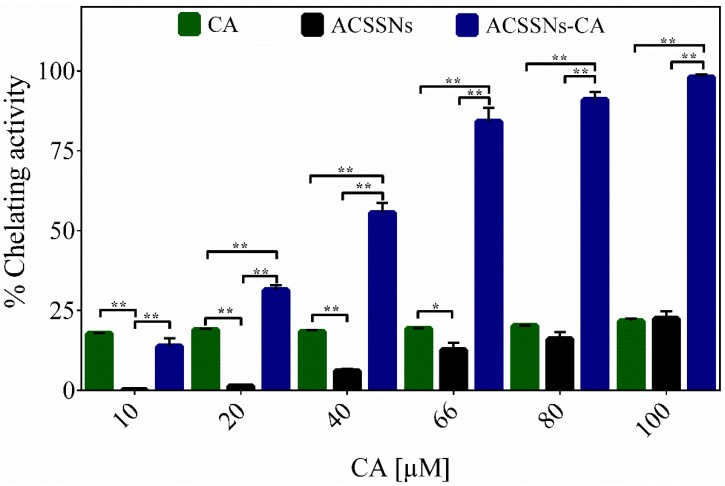
Chelating activity of free caffeic acid (CA), caffeic acid grafted onto core–shell silica nanoparticles (ACSSNs-CA), and caffeic-acid-free nanosphere (ACSSNs). Results are reported as means ± SD (*n* = 3). * *p* < 0.05; ** *p* < 0.001.

The capacity of the nanomaterials as metal chelators was studied, based on their ability to inhibit the colored complex formation of Fe^2+^–ferrozine [42]. Figure 4 shows that as the concentration of antioxidant nanomaterial increased, so did the chelating capacity, expressed as %Fe^2+^-chelating activity. The chelating activity was much higher than that of free caffeic acid. Together with the results for the ACSSNs, which showed a chelating activity between 10% and 20% [43], it can be proposed that the activity was enhanced due to the presence of both new amide bonds and free –NH_2_ groups on the nanoparticle surface, which were favorable conditions for complex formation with the metal through interactions between the metal and the nitrogen in the amide bond, the amino nitrogen, and the amide oxygen [56,57,58].

The deactivation of ^1^O_2_ with polyphenols or other quenchers involves physical quenching (deactivation) and/or chemical (reactivity) processes. The sum of the physical quenching (*k*_Q_) and chemical reaction (*k*_r_) constants correspond to the total quenching rate constant (*k*_q_) [59]. The obtained value of *k*_q_ for ACSSNs-CA was 1.3 × 10^6^ M^−1^·s^−1^, indicating that the nanosystems were potent singlet oxygen quenchers. Despite the *k*_q_ value being lower than those of other molecular antioxidants [59] and antioxidant nanosystems [31], our nanomaterial exhibited a superior quenching activity when compared to free CA (*k*_q_ = 4.6 × 10^5^ M^−1^·s^−1^). The singlet oxygen quenching by CA is related to the electron-donating properties of the catechol moiety. Indeed, a charge-transfer interaction mechanism was proposed by Foley et al. [60]. Nevertheless, the increase in the quenching activity was probably due to the contribution of nanoparticles themselves, as observed by other authors [61,62], mainly as a result of (i) quenching by amino groups on the nanoparticle surface, (ii) quenching by hydrogen-bonded water and silanol groups in the mesopores, and (iii) enhancement of these processes by the increased wall-collision frequency in the silica mesoporous channels [63]. Singlet oxygen deactivation by ACSSNs was determined as a control. The total quenching rate constant of ACSSNs was two orders of magnitude lower than that of ACSSNs-CA, being negligible.

### 3.3. Protection of Nanoparticle-Bound Drugs

As a proof of concept that CA may act as an antioxidant barrier capable of delaying the degradation of a compound incorporated in the nanoparticles, we studied the oxidation of nano-ADPA based on a singlet oxygen generated by an exogenous photosensitizer.

**Figure 5:**
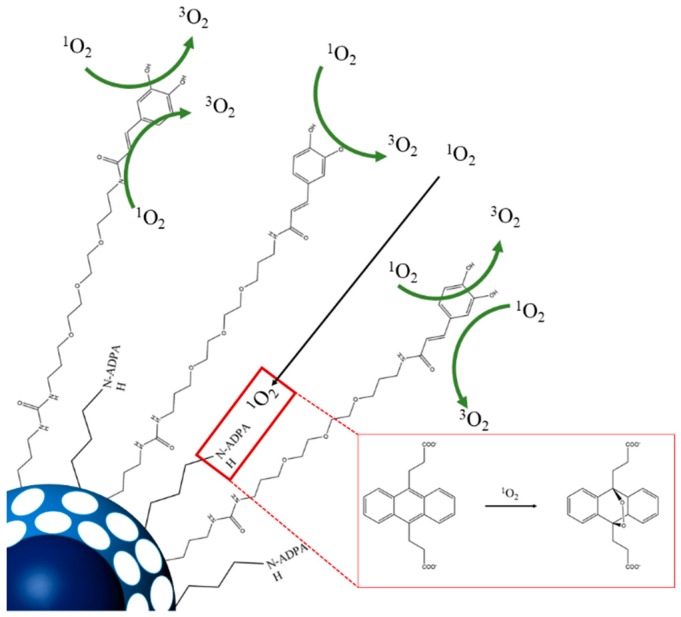
Proposed sketch of protection against singlet oxygen oxidative specie by the antioxidant nanomaterial.

**Figure 6:**
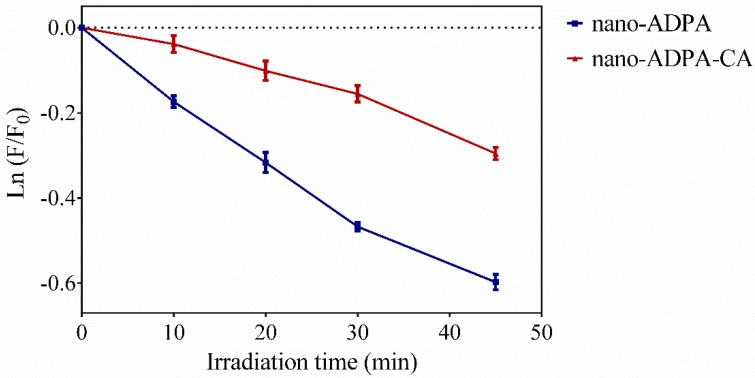
Nano-anthracene dipropionic acid (ADPA) photobleaching (blue line) and nano-ADPA-CA photobleaching (red line) in ethanol. Each point represents the mean ± SD (*n* = 3).

ADPA was linked close to the surface of the nanoparticle, while CA was attached through a longer linker to form a corona acting as a barrier against exogenously generated singlet oxygen (Figure 5). Figure 6 shows the loss of ADPA fluorescence upon irradiation of the photosensitizer rose bengal for both nano-ADPA and nano-ADPA-CA. The rate of nano-ADPA-CA photooxidation was, on average, 66% lower than that of nano-ADPA, which shows that the caffeic acid on the nanoparticle surface could actually protect ADPA from singlet oxygen.

### 3.4. Interaction of the Antioxidant Nanomaterial with BSA

**Figure 7:**
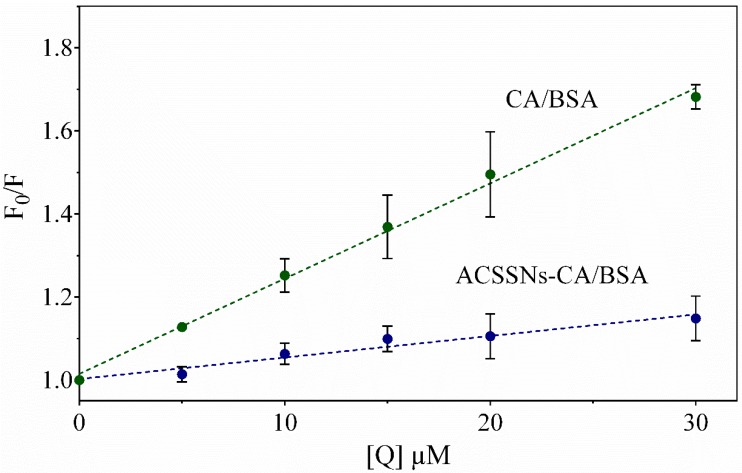
The Stern–Volmer plots of CA/bovine serum albumin (BSA) (green) and ACSSNs-CA/BSA (blue) at 298 K. Each point represents the mean ± SD (*n* = 3).

Fluorescence spectroscopy was used to study the ability of the model protein BSA to form a complex with CA, which would affect its antioxidant capacity in biological milieu [29,64]. The fluorescence intensity of the aromatic residues of BSA showed a significant decrease as free CA was added (Figure 7). On the other hand, the ACSSNs showed a much lower effect, indicating little interaction of BSA with the nanoparticle and its surface groups. In contrast, ACSSNs-CA quenched the BSA fluorescence but the Stern–Volmer constant is 4.4-fold smaller than that of free CA (Figure 7), indicative of the protection afforded by the nanoparticle. This observation was consistent with previous reports from us on related nanosystems [46] and confirmed the adsorption of BSA onto the nanoparticle surface forming a protein corona [65,66].

Table 2 shows the values of *K*_SV_. Assuming a typical value of τ_0_ = 5 ns [48], the values of *k*_q,BSA_ for both the interaction of CA with BSA and ACSSNs-CA with BSA were calculated as 4.6 × 10^12^ M^−1^·s^−1^ and 1.0 × 10^12^ M^−1^·s^−1^, respectively. These values were larger than the diffusion-controlled limit (2 × 10^10^ M^−1^·s^−1^) [67], suggesting that static quenching was the predominant mechanism in the interaction between BSA and free CA, as well as with CA immobilized in nanoparticles. It was reported that electrostatic interactions, hydrogen bonds, and hydrophobic interactions are predominant in the binding of caffeic acid to BSA [68]. In order to determine the binding parameters, the data were adjusted to the double-logarithm model. Table 2 shows the values for *K*_A_ and *n*. As expected, the *K*_A_ for ACSSNs-CA was lower than that for free CA, and *n* was ~1 and ~2 for free CA and ACSSNs-CA, respectively.

## 4. Conclusions

A new hybrid nanocarrier with antioxidant properties was obtained, based on the polyphenol caffeic acid covalently linked onto a core–shell silica nanosphere. An efficient synthetic route and a stable nanosystem were obtained when bare nanoparticles (CSSNs) were firstly functionalized with aminopropyl groups and then caffeic acid was attached through the action of coupling EDC/NHS agents. The radical-scavenging activity of caffeic acid was attenuated in the nanoparticles; however, other antioxidant properties such as Fe^2+^-chelating activity and singlet oxygen quenching were enhanced. In addition, caffeic acid was protected from binding to proteins by the nanoparticle, suggesting that this nanosystem is more likely to maintain the antioxidant activity of caffeic acid in biological media. Finally, caffeic-acid-bound silica nanoparticles were able to delay the degradation due to singlet oxygen of anthracene dipropionic acid incorporated into this nanovehicle. Considering all findings, this work proposes a suitable tool for pharmaceutical and cosmetic industries, whereby antioxidant nanocarriers may increase drug stability due to the protection conferred against oxidative or degradative species.

## Figures and Tables

**Table 1:** Nanoparticle size and zeta potential. All values are expressed as means ± SD (*n* = 3). DLS—dynamic light scattering; PdI—polydispersity index; CA—caffeic acid; TGA—thermogravimetric analysis; CSSNs—core–shell silica nanospheres; ACSSNs—amino-functionalized core–shell silica nanospheres; ACSSNs-CA—core–shell silica nanospheres with immobilized caffeic acid.

Nanoparticle	Diameter by DLS Measurement ^a^ (nm)	PdI	Zeta Potential ^b^ (mV)	mg CA/100 mg Nanoparticles	
				TGA	HPLC
CSSNs	199.1 ± 49.5	0.08 ± 0.02	−32.0 ± 1.0	-	-
ACSSNs	224.5 ± 59.1	0.13 ± 0.03	−3.0 ± 1.0	-	-
ACSSNs-CA	205.7 ± 53.0	0.05 ± 0.03	−22.6 ± 0.2	11.77	12.50

^a^ Determined at 25 °C in ethanol. ^b^ Determined at 25 °C in 1 mM NaCl aqueous solution.

**Table 2:** Quenching and union parameters of CA/bovine serum albumin (BSA) and ACSSNs-CA/BSA, determined at 298 K. Data are presented as means ± SD (*n* = 3).

Compound	*K*_SV_ (× 10^3·^M^−1^)	*k*_q,BSA_ (× 10^12^ M^−1^·s^−1^)	*K*_A_ (× 10^3^·M^−1^)	*n*
CA/BSA	22.9 ± 0.7	4.6 ± 0.1	28.6 ± 4.4	0.94 ± 0.06
ACSSNs-CA/BSA	5.2 ± 0.6	1.0 ± 0.5	6.0 ± 9.8	1.8 ± 0.4
